# Effective reduction of nitrophenols and colorimetric detection of Pb(ii) ions by *Siraitia grosvenorii* fruit extract capped gold nanoparticles†

**DOI:** 10.1039/d1ra01593a

**Published:** 2021-04-26

**Authors:** Van Thuan Le, Truong Giang Duong, Van Tan Le, Thanh Long Phan, Thi Lan Huong Nguyen, Tan Phat Chau, Van-Dat Doan

**Affiliations:** Center for Advanced Chemistry, Institute of Research and Development, Duy Tan University 03 Quang Trung Da Nang City 550000 Vietnam; The Faculty of Environmental and Chemical Engineering, Duy Tan University 03 Quang Trung Da Nang City 550000 Vietnam; Faculty of Chemical Engineering, Industrial University of Ho Chi Minh City 12 Nguyen Van Bao Ho Chi Minh City 700000 Vietnam doanvandat@iuh.edu.vn; Institute of Biotechnology and Food Technology, Industrial University of Ho Chi Minh City Ho Chi Minh City 700000 Vietnam; Institute of Applied Science & Technology, Van Lang University Ho Chi Minh City 700000 Vietnam

## Abstract

This study presents a simple and green approach for the synthesis of *Siraitia grosvenorii* fruit extract capped gold nanoparticles (SG-AuNPs). The SG-AuNPs samples prepared under the optimized conditions were characterized by various techniques (UV-Vis, XRD, FTIR, HR-TEM, EDX, DLS). The biosynthesized nanoparticles were then studied for the reduction of 2-nitrophenol (2-NP) and 3-nitrophenols (3-NP) and for colorimetric detection of Pb^2+^ ions. The characterization results revealed that the crystals of SG-AuNPs were spherical with an average size of 7.5 nm. The FTIR and DLS analyses proved the presence of the biomolecule layer around AuNPs, which played an important role in stabilizing the nanoparticles. The SG-AuNPs showed excellent catalytic activity in the reduction of 3-NP and 2-NP, achieving complete conversion within 14 min. The catalytic process was endothermic and followed pseudo-first-order kinetics. The activation energy was determined to be 10.64 and 26.53 kJ mol^−1^ for 2-NP and 3-NP, respectively. SG-AuNPs maintained high catalytic performance after five recycles. The fabricated material was also found to be highly sensitive and selective to Pb^2+^ ions with the detection limit of 0.018 μM in a linear range of 0–1000 μM. The practicality of the material was validated through the analyses of Pb^2+^ in mimic pond water samples. The developed nanoparticles could find tremendous applications in environmental monitoring.

## Abstract 2

*Siraitia grosvenorii* fruit extract capped AuNPs exhibited excellent catalytic activity in the reduction of nitrophenols and high sensitivity and selectivity for detection of Pb(ii) ions.

## Graphical abstract

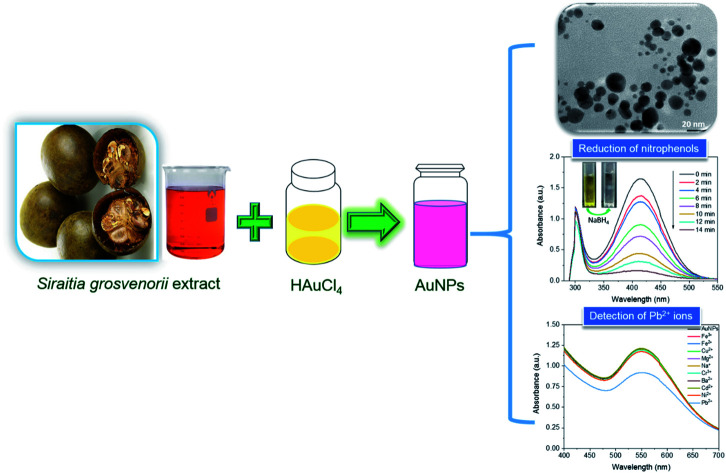

## 1. Introduction

Heavy metals and organic substances are considered as the major pollutants in wastewater, and have caused many serious problems for the environment and human health.^1–3^ Heavy metals like Pb^2+^, Cd^2+^, Hg^2+^, Ni^2+^, and Cr^6+^, even at trace level concentrations, in water bodies have been reported to be potential environmental pollutants.^4^ Delay in detecting and estimating these metal contaminants in biological systems and the aquatic environment could cause several health issues in humans. There are several methods available for detecting and monitoring metal ions in solutions such as electrochemical method, atomic absorption spectrometry, inductively coupled plasma atomic emission spectroscopy, X-ray fluorescence spectrometry, and inductively coupled plasma mass spectrometry. Although those methods provide excellent sensitivity, high accuracy, and multi-element analysis, they have shortcomings associated with the complicated sample preparation, complex and costly instrumentation, and time-consuming procedures.^5^ In recent years, nanomaterial-based colorimetric sensors have been extensively studied for the detection of heavy metal ions in environmental and biological samples owing to their simple operations, cost-effective, highly sensitive and selective detection, and rapid analysis.^6^ This method can allow the direct tracking of target heavy metal ions by the naked eyes through color changes without expensive instruments compared with other methods. With this convenience, the development of various colorimetric sensors for detecting metal contaminants is now of great interest.

Along with heavy metals, organic pollutants containing aromatic structures are often characterized by high stability, non-biodegradability, and high toxicity, leading to various dangerous diseases for humans, including genetic mutations and cancer, when exposed to them.^7^ Particularly, nitrophenols are the most important industrial aromatic compounds widely used in the production of pesticides, pharmaceuticals, dyes, explosives, and rubber. However, these compounds have high toxicity and can potentially damage the nervous central system and the vital organs (*e.g.*, eye, lung, and kidney). Because of that reason, nitrophenols have been listed by the United States Environmental Protection Agency as “Priority Pollutants”, and their concentrations in natural waters are restricted to <10 ng L^−1^.^8^ Therefore, eliminating these harmful pollutants in wastewater is important issue. Various approaches have been applied for removing nitrophenols, such as adsorption, filtration, advanced oxidation processes, and catalytic reduction.^9^ Among these strategies, the reduction of toxic nitrophenols to beneficial aminophenols (AP) by sodium borohydride (NaBH_4_) with metal catalysts has received much attention due to its high effectiveness, low-energy consumption, mild operation condition, and avoiding the secondary pollution.^10^

Gold nanoparticles (AuNPs) have been reported as excellent candidates for the colorimetric detection of heavy metals and catalytic reduction of organic pollutants.^5,9,11–13^ The fascinating optical properties based on surface plasmon resonance (SPR) together with large surface to volume ratios of AuNPs assist highly sensitive and selective detection for heavy metals. The binding of target analyte to AuNPs surface can cause aggregation, resulting in a change of color with the SPR peak shift. This forms the basis of colorimetric detection.^5^ Besides, the unique properties of AuNPs, such as high specific surface area, chemical inertness, reusability, and highly stable dispersions, make them one of the most effective catalysts.^14^ Hitherto, various physical, chemical, and biological (microbes and plant extracts) methods have been used for the synthesis of AuNPs. Among them, green synthesis using extracts from different parts of plants is most preferred as it avoids the use of hazardous chemicals and does not require complicated instruments. Plant extracts contain diverse specific biomolecules (*e.g.*, polysaccharides, proteins, alkaloids, phenolic acids, and flavonoids) that offer the dual roles in both processes of reduction and stabilization of nanoparticles.^15^ Several plants like *Codonopsis pilosula*,^16^*Litsea cubeba*,^17^ cinnamon bark,^18^*Coffea arabica*^19^, *Clitoria ternatea* flower,^20^*etc.*, have been utilized for producing AuNPs. Currently, considerable efforts are being made to find new plant material for the eco-friendly synthesis of AuNPs.

*Siraitia grosvenorii* (SG), a species of the Cucurbitaceae family, has been cultivated widely in south China, Indonesia, and Vietnam.^21^ Fruits of SG plant are used not only as a food ingredient but also as a traditional medicine for the treatment of laryngitis, diabetes, bronchitis, and gastrointestinal diseases.^22^ Studies showed that the SG fruit extract constitutes a rich source of triterpenoids, glycosides, flavonoids, polysaccharides, and proteins. These phytochemicals, as mentioned above, can act as reducing and capping agents for the preparation of AuNPs.

In the present study, we report a simple and green strategy for the rapid synthesis of AuNPs using SG fruit extract. The prepared SG-AuNPs were characterized comprehensively using various instrumental techniques before evaluating their application in the catalytic reduction of 2-nitrophenol (2-NP) and 3-nitrophenol (3-NP) and colorimetric detection of Pb(ii). The catalytic efficiency, reusability, kinetic behavior, and activation energy for the reduction of 2-NP and 3-NP by SG-AuNPs were determined. The sensitivity and selectivity of SG-AuNPs toward several metal ions were also tested. Furthermore, the mechanism and limits of detection (LOD) for Pb(ii) ions were elucidated. Finally, the proposed method was also applied for the Pb(ii) determination in simulated pond water.

## 2. Materials and methods

### 2.1. Materials

Tetrachloroauric acid trihydrate (HAuCl_4_·3H_2_O, ≥99.9%) of ACS reagent grade was supplied by Acros Organics (Belgium). Sodium borohydride (NaBH_4_, ≥98.0%), 2-nitrophenol (2-NP, C_6_H_5_NO_3_, ≥98.0%), 3-nitrophenol (3-NP, C_6_H_5_NO_3_, ≥98.0%), and all metal salts were commercial products from Merck (Singapore). The SG fruits were collected from the northern highlands of Vietnam in September and dried naturally under sunlight until the humidity was about 12%. Deionized water was used for all experiments and the necessary glassware should be rinsed and dried well before use.

### 2.2. Preparation of SG extract

Before the extraction, the peel and seeds of SG dried fruits were removed. The flesh of SG fruits (5 g) was boiled gently in distilled water (400 mL) on a thermomagnetic stirrer with reflux for 1 h. The obtained light brown extract was cooled down to room temperature. The remaining solids in the SG extract were separated by filtration with Whatman filter paper no. 1. The transparent extract was then ready for further synthesis process of SG-AuNPs.

### 2.3. Biosynthesis of AuNPs

The synthesis procedure of biogenic SG-AuNPs from HAuCl_4_ solution and aqueous SG fruit extract was similar to that described in our previous work.^16^ Briefly, the SG fruit extract was mixed with HAuCl_4_ solution at a volume ratio of 1 : 10. The obtained mixture was vigorously stirred in the dark to protect the newly formed nanoparticles from any possible photochemical reaction. After the synthesis process, the color of the solution was changed due to the SPR phenomenon, which can be a visual indication of the SG-AuNPs formation. The metallic ion concentration, reaction temperature, and reaction time were optimized. The colloidal SG-AuNPs solution synthesized at the optimal conditions were further used for the studies of the morphology and structure, nitrophenols degradation, and Pb(ii) detection.

### 2.4. Characterization of AuNPs

Powder X-ray diffraction (XRD) was performed on a Shimadzu 6100 X-ray diffractometer (Japan) with CuKα radiation at the wavelength of 1.5406 nm, accelerating voltage of 40 kV, current of 30 mA, scan speed of 0.05° s^−1^, step size of 0.02°, and 2*θ* range from 10° to 80°. Fourier transform infrared (FTIR) spectroscopy was conducted on a Bruker Tensor 27 spectrometer (Germany) for detecting functional groups in the SG fruit dried extract and SG-AuNPs. High-resolution transmission electron microscopy (HR-TEM) was carried out on a JEM Jeol 2100 (Japan) to investigate the morphology and particle size of SG-AuNPs in colloidal solution form. Selected area electron diffraction (SAED) equipped on the HR-TEM was applied to confirm the crystalline nature of SG-AuNPs. Energy-dispersive X-ray (EDX) spectroscopy integrated with the HR-TEM was also used to acquire EDX spectrum and stem mapping image. Finally, the dynamic light scattering (DLS) and zeta potential measurements of SG-AuNPs were operated on a Horiba SZ-100 (Japan).

### 2.5. Catalytic reduction experiments

The catalytic performance of SG-AuNPs was examined for the reduction reaction of 2-NP and 3-NP with NaBH_4_ solution *via* UV-Vis measurements. The reaction process was similarly described in our previous study.^17^ Briefly, 2.5 mL 1.0 mM nitrophenols solution was mixed with freshly prepared 0.5 mL 0.1 M NaBH_4_ solution at room temperature (25 ± 1 °C) in a standard cuvette with an optical path length of 1.0 cm. Next, 3 mg of SG-AuNPs was added, and UV-Vis measurements were then performed at the wavelength ranged from 250 to 550 nm on the Cary 60 UV-Vis spectrophotometer (Agilent, USA). To evaluate the reusability of SG-AuNPs, the reduction of nitrophenols was performed repeatedly in five consecutive cycles in the presence of reused SG-AuNPs. After each cycle, the biogenic SG-AuNPs were separated from the reaction solution by ultracentrifuge rotating and washed with ethanol and then distilled water several times before reuse.

### 2.6. Colorimetric detection of Pb(ii)

In this study, the biosynthesized SG-AuNPs was utilized to examine its colorimetric sensing feature for various metal ions, including Pb^2+^, Ni^2+^, Cd^2+^, Ba^2+^, Cr^3+^, Na^+^, Mg^2+^, Cu^2+^, Fe^2+^, and Fe^3+^. Firstly, the colloidal solution of SG-AuNPs (400 μL) was added separately into a series of metal ions at the same concentration (200 μL, 1000 μM). Then, UV-Vis measurements were performed in a range of 400 to 700 nm along with the change in color of SG-AuNPs solution. The procedure for selectively colorimetric quantification of Pb^2+^ was conducted as follows. A solution of SG-AuNPs (400 μL) was added into Pb(ii) solutions (200 μL) with the concentration range of 0 ÷ 5000 μM. The characteristic SPR peak of SG-AuNPs was then recorded, as well as the detectable range and linear aggression equation were established. The ability to apply the method in practice was confirmed using mimic pond water prepared from aqueous solutions of NaCl (0.9%), BaCl_2_ (0.4%), and FeCl_3_ (0.6%).^23^ Pb(NO_3_)_2_ salt was added to obtain solutions with Pb(ii) concentration of 20–350 μM. The amount of Pb(ii) in the as-prepared mimic pond water was rechecked by using the SG-AuNPs.

## 3. Results and discussion

### 3.1. Optimization of synthesis parameters

**Fig. 1:**
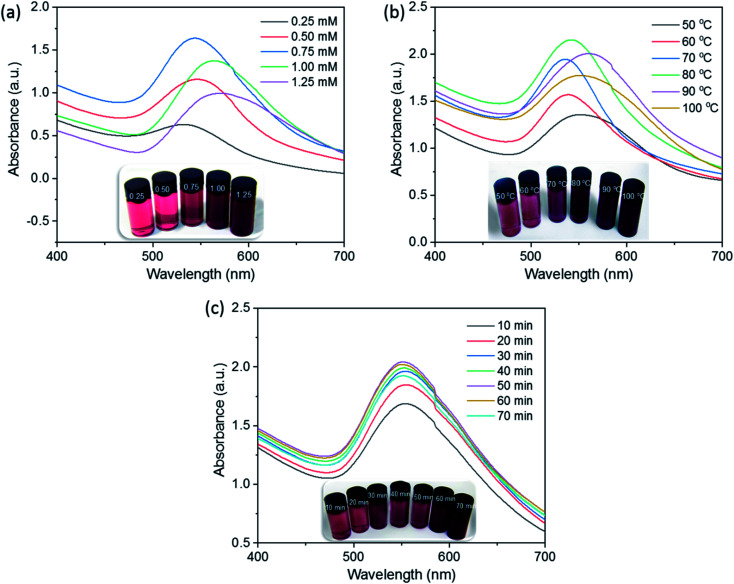
Effect of Au^3+^ concentration (a), reaction temperature (b), and reaction time (c) on the formation of SG-AuNPs.

The optimization of synthesis parameters is needed to find the suitable condition for producing qualified AuNPs. In this study, the principal parameters, including gold ion concentration (0.25–1.25 mM), reaction temperature (50–100 °C), and reaction time (10–70 min), were investigated using UV-Vis absorption spectra. The effects of these factors on the SG-AuNPs formation were traced by the change in intensity and position of the SPR peak. The experimental results are shown in Fig. 1.

Fig. 1a shows the effect of the gold ion concentration on the AuNPs synthesis at the reaction temperature and time of 50 °C and 50 min, respectively. It can be seen that the intensity of the SPR peak for AuNPs at 540 nm increased as the concentration of gold ions increased from 0.25 to 0.75 mM; and then decreased significantly with further rising the amount of gold ions rate up to 1.25 mM. The decrease in intensity of the SPR peak was probably due to the coagulation of the formed AuNPs solution.^16^ The high concentration of gold ions used (>0.75 mM) also led to the shifting of the SPR peak from 540 nm to 560 nm, reflecting that varying the concentration of Au^3+^ solution affected the shape and size of the synthesized AuNPs.^24^

Further experiment tests on the effect of temperature on the synthesis of AuNPs were performed at the range of 50–100 °C while keeping the gold ion concentration at 0.75 mM and reaction time at 50 min. The results presented in Fig. 1b show that with increasing the reaction temperature, the AuNPs content increased gradually and achieved a maximum value at 80 °C. At higher temperatures, rapid movement of AuNPs could increase effective collisions between them, which led to the formation of larger nanoparticles and promoted coagulation, reducing the intensity of the absorption peak.^23^ The displacement of the SPR peak recognized during the survey process indicated the significant influence of reaction temperature on the size and morphology of AuNPs.

Finally, for optimizing the reaction time, the reaction of Au^3+^ solution (0.75 mM) with the SG fruit extract was carried out at 80 °C, and the UV-Vis measurements were performed every 10 min. As presented in Fig. 1c, all spectra exhibited the SPR peak around 540 nm without significant peak shifts, implying no remarkable change in size and shape of developed AuNPs over reaction time. The intensity of the SPR band was low for the first 10 min of reaction because of the slow conversion of Au^3+^ to AuNPs, then it notably increased with the increase of the reaction time and reached a maximum after 50 min. When the synthesis time was extended to more than 50 min, a slightly decrease in the SPR peak intensity was observed. This phenomenon might be due to the aggregation of nanoparticles caused by the excess of thermal energy provided. Based on the obtained results, the optimal gold ion concentration, reaction temperature, and reaction time were chosen at 0.75 mM, 80 °C, and 50 min, respectively.

### 3.2. Characterization of biogenic AuNPs

**Fig. 2:**
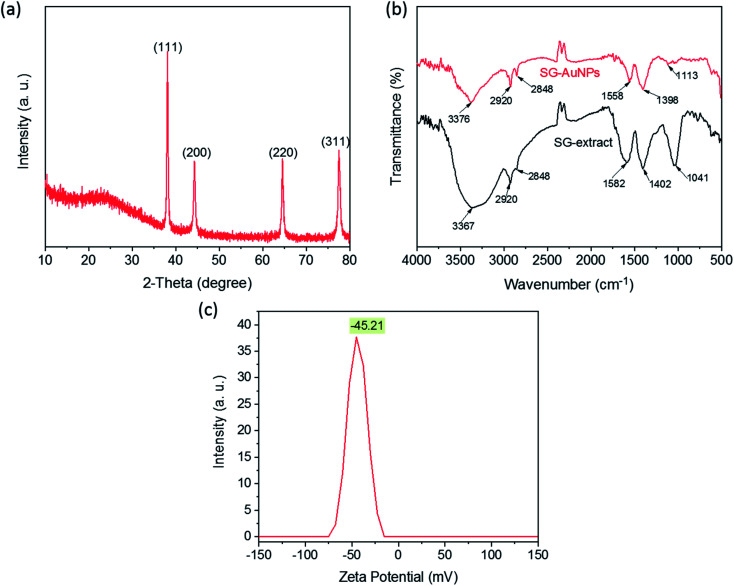
XRD pattern (a), FTIR spectra (b), and zeta potential of SG-AuNPs (c).

The crystal phase composition of AuNPs synthesized under optimized conditions was characterized by the XRD pattern, as shown in Fig. 2. The XRD pattern displays four prominent diffraction peaks at 2*θ* of 38.02°, 44.42°, 64.56°, and 75.53° corresponding to (111), (200), (220), and (311) planes, confirming the face-centered crystalline cubic structure of AuNPs (ICDD PDF No. 00-004-0784). The broad peak observed around 2*θ* of 24° could be related to amorphous phases of the organic compounds surrounding AuNPs. A similar observation was also reported in previous studies.^25,26^ Furthermore, the ratio between the intensity of the (111) peak and other peaks was high, suggesting that the (111) plane was the predominant growth direction in the crystal of AuNPs.^17^

The FTIR measurements were taken to identify surface functional groups of the SG-AuNPs sample and dried SG fruit extract. As illustrated in Fig. 2b, the FTIR spectrum of SG-AuNPs exhibited a high similarity in absorption bands with the SG fruit extract, indicating the presence of the organic layer that surrounded AuNPs. Notably, the significant shifts of some diffraction peaks for SG-AuNPs were also observed. These shifts can be caused by the influence of the nearby gold surface.^27^ The main functional groups of SG fruit extract were defined as bellows. The strong signal at 3367 cm^−1^ was attributed to the stretching vibrations of O–H group arising from glycosides, flavonoids, and polysaccharides in the SG fruit extract.^25^ The absorption bands at 2920 and 2848 cm^−1^ were assigned to the stretching vibrations C–H from –CH_3_ and –CH_2_ groups. The peaks around 1582 and 1041 cm^−1^ were related to the asymmetric –COO stretching. The absorption signal appeared at 1402 cm^−1^ was associated with the –C=C stretching vibration of aromatic rings.^17^ It is these phytoconstituents (flavonoids and phenolic compounds) that reduced Au^3+^ ions into Au^0^ by oxidizing hydroxyl (R–OH) to carbonyl groups (R–C=O) or transfer their π-electrons.^15,28^ The biomolecules present in the aqueous extract of SG fruit were further adsorbed on the AuNPs surface to form an organic protective layer responsible for the stabilization of the synthesized AuNPs. This also was proved by the stability of SG-AuNPs in aqueous solution, which was assessed by its zeta potential, as provided in Fig. 2c. As expected, the zeta potential value of SG-AuNPs solution at pH 5.5 was quite high (−45.21 mV), suggesting its excellent stability for a long time.

**Fig. 3:**
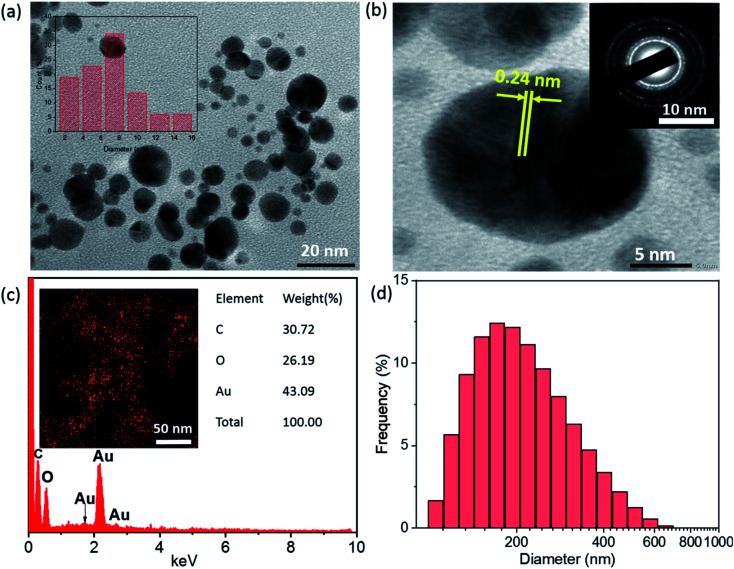
TEM image (a), HR-TEM image (b), SEAD (insert of b), EDX spectrum (c), EDX element mapping (insert of c), and DLS diagram (d) of SG-AuNPs.

The size, shape, and crystalline nature of SG-AuNPs were established by TEM image (Fig. 3a), HR-TEM (Fig. 3b) and SAED pattern (insert in Fig. 3b). As can be noticed from the TEM image, the biosynthesized SG-AuNPs were presented in a spherical shape with a size ranged between 2.5 and 15 nm. Meanwhile, the particle-size distribution obtained from TEM images (insert in Fig. 3a) indicated that the average size of SG-AuNPs was found to be 7.5 nm. The SAED pattern showed four bright circular rings corresponding to the (1 1 1), (2 0 0), (2 2 0), and (3 1 1) planes of AuNPs, revealing the crystalline nature of the synthesized material. The HR-TEM image manifested the lattice fringe of 0.24 nm, which was in agreement with those of AuNPs prepared using an aqueous extract of *Codonopsis pilosula* roots.^16^

Fig. 3c displays the EDX spectrum and element mapping for the SG-AuNPs sample. The EDX spectrum revealed that SG-AuNPs were mainly composed of Au (43.09%), C (30.72%), and O (26.19%). In addition, the presence and distribution of Au can be clarified by the mapping pattern as depicted in the insert of Fig. 3c. The existence of other elements such as C and O in the SG-AuNPs sample was derived from the encapsulating biomolecules. Further, DLS measurements were carried out to determine the size of SG-AuNPs in aqueous solution. The DLS diagram (Fig. 3d) showed the particle size distribution in the range of 30–650 nm with an average diameter centered at 150 nm, which was much higher than that from the TEM analysis. The variation in DLS and TEM results can be explained by the fact that the DLS analysis measured hydrodynamic diameter of particles including the biomolecules layer around AuNPs, which was not observed by the TEM technique.^29^ Thus, all characterization results proved the success of the SG-AuNPs synthesis using SG fruit extract.

### 3.3. Catalytic activity of AuNPs for reduction of nitrophenols

**Fig. 4:**
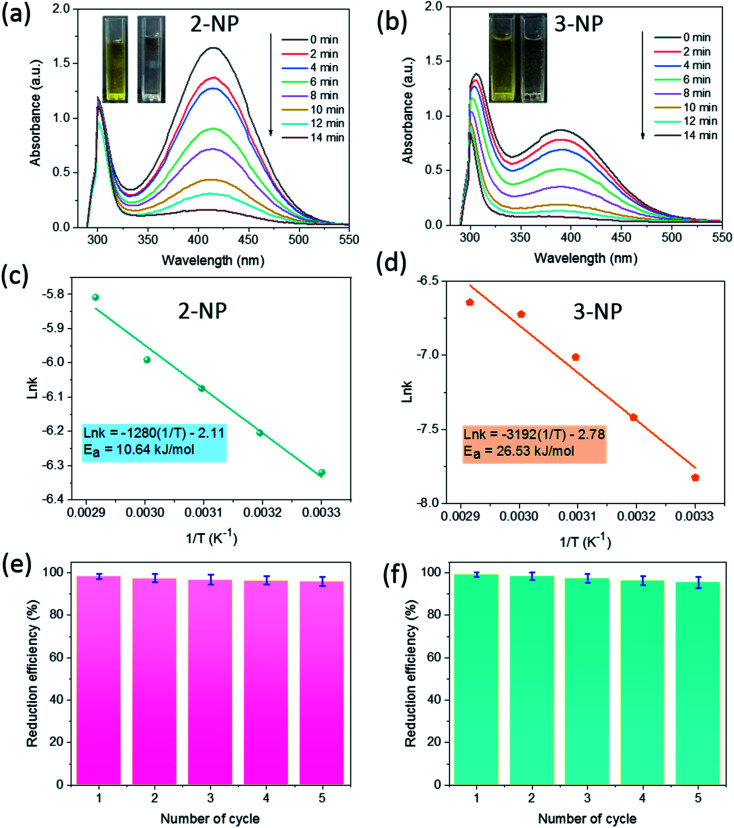
UV-Vis spectra (a) and (b), Arrhenius plots of ln *k versus* 1/*T* (c) and (d), and reusability (e) and (f) of SG-AuNPs for catalytic reduction of 2-NP and 3-NP.

The catalytic activity of SG-AuNPs was measured through the reduction reaction of 2-NP and 3-NP with NaBH_4_, which can be occurred by the electron transfer process from BH_4_^−^ donor to nitrophenols acceptor *via* the AuNPs surface.^23^ The UV-Vis absorption spectra during the reduction of 2-NP and 3-NP by NaBH_4_ without and with the SG-AuNPs catalyst are presented in Fig. S3a, b† and 4a, b, respectively. As shown in Fig. S3 (ESI†), the reduction was negligible in the absence of the catalyst, only about 5% of 2-NP and 3-NP was reduced within 60 min. Meanwhile, adding SG-AuNPs significantly increased the conversion of 3-NP and 2-NP into 3-AP and 2-AP; the reduction was completed in 14 min (Fig. 4a and b). The complete reduction of the pollutants was also observed through color change with bleaching of the yellow color (inserts in Fig. 4a and b).

The catalytic reactions were further carried out at the temperature range of 30–70 °C (see Fig. S1 and S2 in ESI†) to obtain insights into the reaction rate and thermodynamic parameters such as activation energy (*E*_a_), enthalpy, (Δ*H*), entropy (Δ*S*), and Gibbs energy (Δ*G*). Because the reaction used an excessive concentration of NaBH_4_ and a very small amount of SG-AuNPs catalyst, the kinetics for reduction of these contaminants can be well described by pseudo-first-order equation (Langmuir–Hinshelwood model) (eqn (1)), whose rate constant can be determined from the plot of ln *A*_*t*_/*A*_0_*versus* reaction time.^14^ Next, the Arrhenius equation (eqn (2)) was applied to calculate the activation energy, while the enthalpy, entropy, and Gibbs energy of activation were determined using eqn (3)–(5), respectively.^30^1
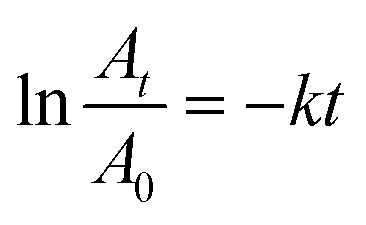
2
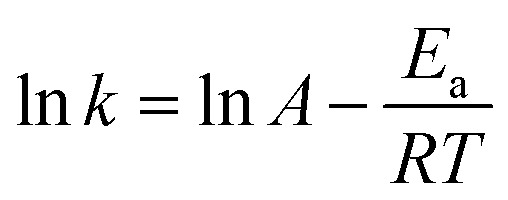
3
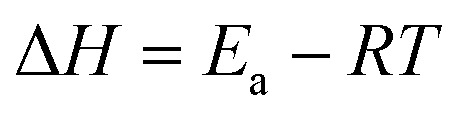
4
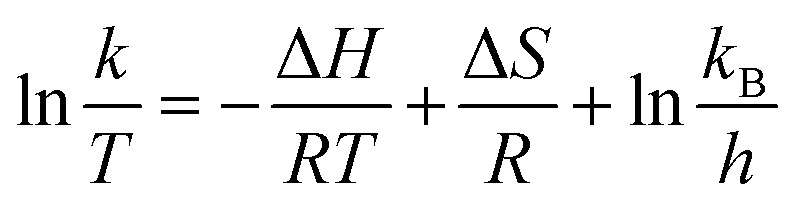
5
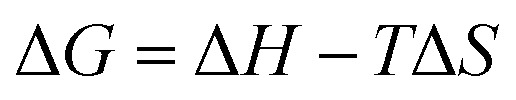
where *A*_*t*_ and *A*_0_ are the absorbance of nitrophenols solution at time *t* and the initial time, respectively; *k* (s^−1^) is the rate constant; *t* (s) is the reaction time; *A* is the pre-exponential factor; *R* (8.314 J K^−1^ mol^−1^) is the ideal gas constant; *T* (K) is the absolute temperature; *k*_B_ is the Boltzmann constant (1.38 × 10^−23^ J K^−1^); and *h* is Planck's constant (*h* = 6.63 × 10^−34^ J s^−1^).

The results given in Table 1 indicated that the rate constant increased with increasing temperature. The reason could be related to the increase in kinetic energy of contaminant molecules, enhancing the contact frequency between them and the catalyst, which resulted in the rapid reaction rate. A similar observation was also reported by other researchers using metal nanoparticles in reducing nitrophenols.^30,31^ In addition, the correlation coefficients from the plots of ln *A*_*t*_/*A*_0_*versus t* for reduction of both 2-NP and 3-NP were higher than 0.95, implying that kinetics of the catalytic reaction were well described by the pseudo-first-order Langmuir–Hinshelwood model. All values of activation enthalpy in the investigated temperature range were positive, indicating the endothermic nature of the catalytic process, while the positive Δ*G* values suggested non-spontaneous reaction. The negative values of Δ*S* demonstrated the more stability of the activated complex.^32^ Meanwhile, the activation energy was calculated to be 10.64 and 26.53 kJ mol^−1^ for 2-NP and 3-NP, respectively. These values are significantly lower than that for the reduction of 2-NP (39.86 kJ mol^−1^) and 3-NP (36.87 kJ mol^−1^) by NaBH_4_ using zerovalent copper-nanopolyaniline-nanozirconium silicate catalyst,^32^ revealing the high effectiveness of the synthesized SG-AuNPs. It can also be noticed from Table 1 that the *E*_a_ and Δ*H* values among 2-NP and 3-NP were quite different, while the corresponding values of ΔG were less variable. The observed behavior could be explained by the known fact, which claims that the free energy of activation Δ*G* and not *E*_a_ or Δ*H* determine the reaction rate.^32^ In addition, the SG-AuNPs biosynthesized by the SG fruit extract also exhibited an acceptable catalytic activity in comparison with other reported AuNPs systems (see Table S1 in ESI†).

**Table 1:** Thermodynamic parameters for degradation of 2-NP and 3-NP by SG-AuNPs

Nitroderivative	Temperature (K)	Rate constant (s^−1^)	*R* ^2^	*E*_a_ (kJ mol^−1^)	Δ*H* (kJ mol^−1^)	Δ*S* (J mol^−1^ K^−1^)	Δ*G* (kJ mol^−1^)
2-NP	303	0.0018	0.956	10.64	8.12	−271.12	90.27
	313	0.0021	0.974		8.04	−271.23	92.93
	323	0.0023	0.968		7.95	−271.79	95.74
	333	0.0025	0.982		7.87	−272.34	98.56
	343	0.0030	0.979		7.79	−271.99	101.08
3-NP	303	0.0004	0.954	26.53	24.01	−231.18	94.06
	313	0.0006	0.991		23.93	−230.88	96.19
	323	0.0009	0.967		23.84	−230.39	98.26
	333	0.0012	0.982		23.76	−230.72	100.59
	343	0.0014	0.969		23.67	−232.01	103.26

Recyclability is an important criterion that determines the applicability of a catalyst. To explore the recyclability of SG-AuNPs, the catalyst was reused for five successive cycles under the same operating conditions (see in Section 2.5), and the degradation efficiency was determined using a ratio between the absorbance values at 14 min and at initial time. The results shown in Fig. 4e and f indicated that the mean performance for reducing both 2-NP and 3-NP gradually decreased with increasing recycle number, which was probably due to the loss of the catalyst during the reuse process. However, SG-AuNPs still maintained a relatively high efficiency of more than 95% after five recycles, demonstrating its excellent reusability. Besides, the morphology of SG-AuNPs after recycling was also evaluated by the TEM analysis. As shown in Fig. S4 (ESI†), the shape and size of SG-AuNPs in general had not changed much compared to before use. Nevertheless, a slight aggregation between particles was observed, which could contribute to the reduction in the catalytic efficiency of SG-AuNPs during reuse.

### 3.4. Detection of Pb(ii) ions

**Fig. 5:**
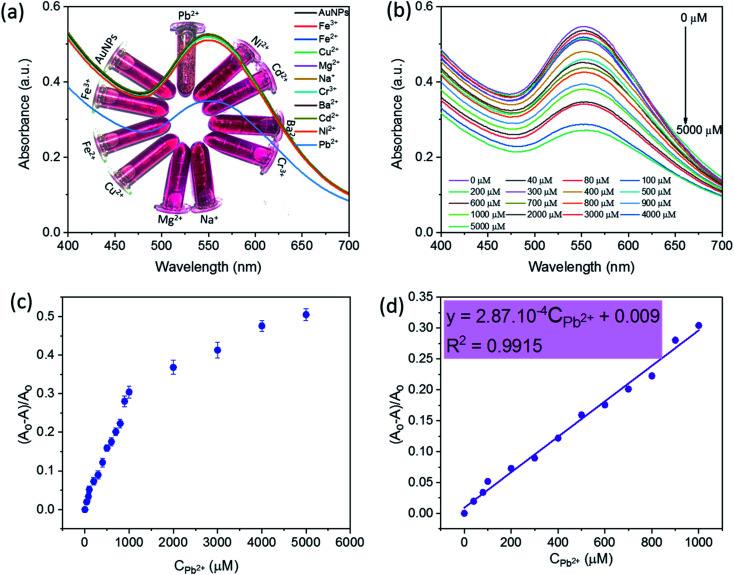
UV-Vis spectra of SG-AuNPs solution with different metal ions (a) and at the Pb(ii) concentration range of 0–5000 μM (b), and plot of sensitivity *versus* relative Pb(ii) concentrations ranged from 0–5000 μM (c) and from 0–1000 μM (d).

The detection ability of SG-AgNPs was tested with various metal ions including Pb^2+^, Ni^2+^, Cd^2+^, Ba^2+^, Cr^3+^, Na^+^, Mg^2+^, Cu^2+^, Fe^2+^, and Fe^3+^ at an unaltered concentration of 1000 μM metal ion solution (200 μL). The change in solution color and absorbance intensity of the SPR band was recorded and displayed in Fig. 5a. Obviously, adding metal ions (Ni^2+^, Cd^2+^, Ba^2+^, Cr^3+^, Na^+^, Mg^2+^, Cu^2+^, Fe^2+^, and Fe^3+^) caused an unremarkable change in the intensity of the SPR band and the color of SG-AuNPs solution. In contrast, the intensity of the SPR band was significantly decreased when Pb^2+^ ions were added. The main reason related to the observed phenomenon can be that these metal ions exhibited much weaker chelating ability with SG-AuNPs than Pb^2+^, consequently demonstrating the high selectivity of SG-AuNPs toward Pb^2+^.^33^

The quantitative detection of Pb^2+^ was investigated by varying the concentrations of Pb^2+^ ions to the colloidal SG-AuNPs solution at similar conditions. The UV-Vis spectra of Pb(ii)–SG-AuNPs at different Pb^2+^ concentrations from 0 μM to 5000 μM are illustrated in Fig. 5b. It can be seen that the maximum wavelength value of the SPR peak was almost unchanged in the studied range of concentration. Meanwhile, the intensity of the SPR peak gradually reduced with increasing concentration of Pb^2+^. The intensity decrease was caused by the aggregation of the particles, which was also recognized in the earlier reported studies.^12,33,34^

Fig. 5c shows the plot of relative values sensitivity ((*A*_0_ − *A*)/*A*_0_, where *A*_0_ and *A* are the SPR absorbance of SG-AuNPs at zero and relative Pb^2+^ concentrations, respectively) against the concentration of Pb^2+^. The result displayed the non-linear effect of various Pb^2+^ concentrations on the relative sensitivity over the entire investigated concentration range of 0–5000 μM. However, in the region of 0–1000 μM, the fitted curve exhibited a linear behavior, which is described by the regression equation (*A*_0_ − *A*)/*A*_0_ = 2.87 × 10^−4^ CPb_^2+^_ + 0.009 with a correlation coefficient of 0.9915 (Fig. 5d). Besides, the LOD that determined by 3SD (SD is standard deviation) was 0.018 μM (or 3.7 μg L^−1^), which meets the mandated upper limit of 10 μg L^−1^ and 15 μg L^−1^ for Pb^2+^ in drinking water by the World Health Organization and the U.S. Environmental Protection Agency, respectively.^35^ More importantly, the SG-AuNPs probe provided the linear range and LOD value competitive with other colorimetric methods for the analysis of Pb^2+^ as listed in Table 2. Thus, the obtained attainments confirmed an excellent quantification of Pb^2+^ at low concentrations assayed by SG-AuNPs.

**Table 2:** Comparison of different colorimetric detection methods toward Pb(ii)

Materials	LOD (μM)	Linear range (μM)	Ref.
AgNPs-mussel-inspired protein	94	60–160	36
Valine capped AuNPs	30.5	1–100	34
Gallic acid-capped AuNPs	0.01	0.01–1	37
Maleic acid-AuNPs	0.002	0–0.048	11
l-Tyrosine-AuNPs	0.016	0.02–0.1	12
Graphene/Fe_3_O_4_-AuNPs	0.003	0.005–1.5	38
SG-AuNPs	0.018	0–1000	This study

**Fig. 6:**
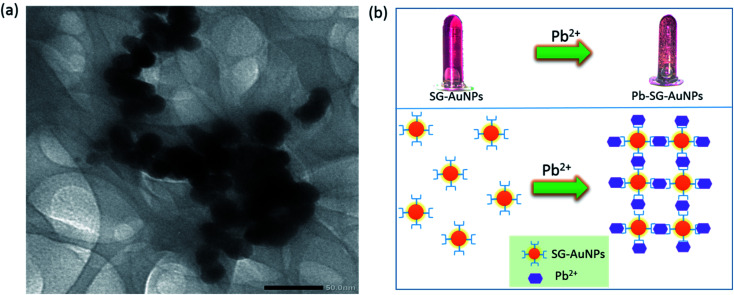
TEM image of SG-AuNPs after mixing with 100 μM Pb(ii) ions (a), and proposed mechanism for colorimetric detection of Pb(ii) (b).

The mechanism of detecting heavy metal ions by AuNPs is generally based on monitoring changes of the SPR peak (position and intensity) and the color of the mixed solution. These changes are the results of the aggregation/agglomeration of nanoparticles with the target-analyte *via* capping functional groups attached to the AuNPs surface.^34^ The analysis of TEM images for SG-AuNPs before and after adding Pb^2+^ clearly revealed the agglomeration of biosynthesized nanoparticles. Indeed, before treatment with Pb^2+^ ions, SG-AuNPs were presented as individual separated particles (Fig. 3a). The good dispersion of particles was attributed to an efficient electrostatic barrier around the particles confirmed by its high value of zeta potential. The negative charge of SG-AuNPs was provided by hydroxyl and carboxyl groups from the SG extract, which offered a good electrostatic repulsion against the vander-Waals attraction between the nanoparticles, thus prohibiting their agglomeration. When mixing SG-AuNPs with 100 μM Pb(ii) ions, the nanoparticles quickly agglomerated, as shown in Fig. 6a. The agglomeration of SG-AuNPs could be ascribed to the chelating interaction of Pb^2+^ ions with hydroxyl and carboxyl groups, which resulted in the destabilization of net negative charge on nanoparticles surface, consequently causing agglomeration of particles.^12,34^ Based on the results of the present study and previous studies, the possible mechanism for colorimetric detection of Pb^2+^ ions using SG-AuNPs was proposed and illustrated in Fig. 6b.

To evaluate the applicability of the proposed detection method, the developed probe was applied to determine Pb^2+^ in mimic pond water containing NaCl (0.9%), BaCl_2_ (0.4%), and FeCl_3_ (0.6%). A calculated amount of Pb(NO_3_)_2_ was added into the mimic pond water to achieve Pb^2+^ concentrations of 20–350 μM. The target-analyte concentration in the spiked water sample was further tested by the SG-AuNPs assay. The measurement results are summarized in Table 3. No noticeable differences between the spiked and determined concentrations were observed, and the recoveries of the tested samples ranged from 99.3% to 106.5%. Therefore, these results demonstrated the utility of the developed nanosensor for the accurate detection of Pb^2+^ ions in water samples.

**Table 3:** Determination of Pb^2+^ in mimic pond water samples using SG-AuNPs

Samples	Spiked concentration (μM)	Determined concentration (μM)	Recovery (%)
1	20	21.3 ± 0.9	106.5
2	50	52.2 ± 1.2	105.0
3	75	74.5 ± 1.6	99.3
4	150	153.6 ± 1.5	102.4
5	350	352.1 ± 2.1	100.6

## 4. Conclusions

The simple and green route was successfully developed for the synthesis of AuNPs using SG fruit extract as a reducing and stabilizing agent. The gold ion concentration, reaction temperature, and reaction time were optimized at 0.75 mM, 80 °C, and 50 min, respectively, to achieve the best nanoparticles. The prepared nanoparticles were in crystal structure with an average size of 7.5 nm and were stable in an aqueous medium for over a month. SG-AuNPs exhibited high catalytic activity and recyclability for reduction of nitrophenols (2-NP and 3-NP), reaching complete conversion within 14 min and maintaining the efficiency of more than 95% after five recycles. Besides, the synthesized SG-AuNPs also showed excellent sensitivity and selectivity for Pb^2+^ compared to other tested metals ions. Pb^2+^ ions could be accurately detected with LOD of 0.018 μM by the SG-AuNPs probe. The metal ion induced aggregation of SG-AuNPs was established as the main mechanism of the detection assay. The tests in mimic pond water samples validated the practicality of the proposed method for analysis of Pb^2+^ ion in real water systems. The overall results indicated that the developed SG-AuNPs material could be used as an effective catalyst and sensing system for the reduction of nitrophenols and the detection of heavy metal ions in aqueous medium.

## Conflicts of interest

There are no conflicts to declare.

## Supplementary Material

RA-011-D1RA01593A-s001
