# Supplementary material for: Effective reduction of nitrophenols and colorimetric detection of Pb(ii) ions by Siraitia grosvenorii fruit extract capped gold nanoparticles
Source: RSC Adv. 2021 Apr 26;11(25):15438–48. doi: 10.1039/d1ra01593a (PMC8698254; doi:10.1039/d1ra01593a)
Supplement: RA-011-D1RA01593A-s001 [file RA-011-D1RA01593A-s001.pdf]

Electronic Supplementary Material (ESI) for RSC Advances.
This journal is © The Royal Society of Chemistry 2021

SUPPLEMENTARY MATERIAL
FOR
Effective reduction of nitrophenols and colorimetric detection of Pb(II) ions by *Siraitia grosvenorii* fruit extract capped gold nanoparticles

Van Thuan Le[1,2], Truong Giang Duong[3], Van Tan Le[3], Thanh Long Phan[3], Thi Lan Huong Nguyen[4], Tan Phat Chau[5], Van-Dat Doan[3,*]

[1]*Center for Advanced Chemistry, Institute of Research and Development, Duy Tan University, 03 Quang Trung, Da Nang, 550000, Vietnam*

[2]*The Faculty of Environmental and Chemical Engineering, Duy Tan University, 03 Quang Trung, Da Nang, 550000, Vietnam*

[3]*Faculty of Chemical Engineering, Industrial University of Ho Chi Minh City, 12 Nguyen Van Bao, Ho Chi Minh, 700000, Vietnam*

[4]*Institute of Biotechnology and Food Technology, Industrial university of Ho Chi Minh City, Ho Chi Minh, 700000, Vietnam*

[5]*Institute of Applied Science & Technology, Van Lang University, Ho Chi Minh 700000, Vietnam*

***Corresponding author:** Van-Dat Doan, Faculty of Chemical Engineering, Industrial University of Ho Chi Minh City, Ho Chi Minh City, Vietnam.

Email: doanvandat@iuh.edu.vn . ORCID: 0000-0003-1838-1836

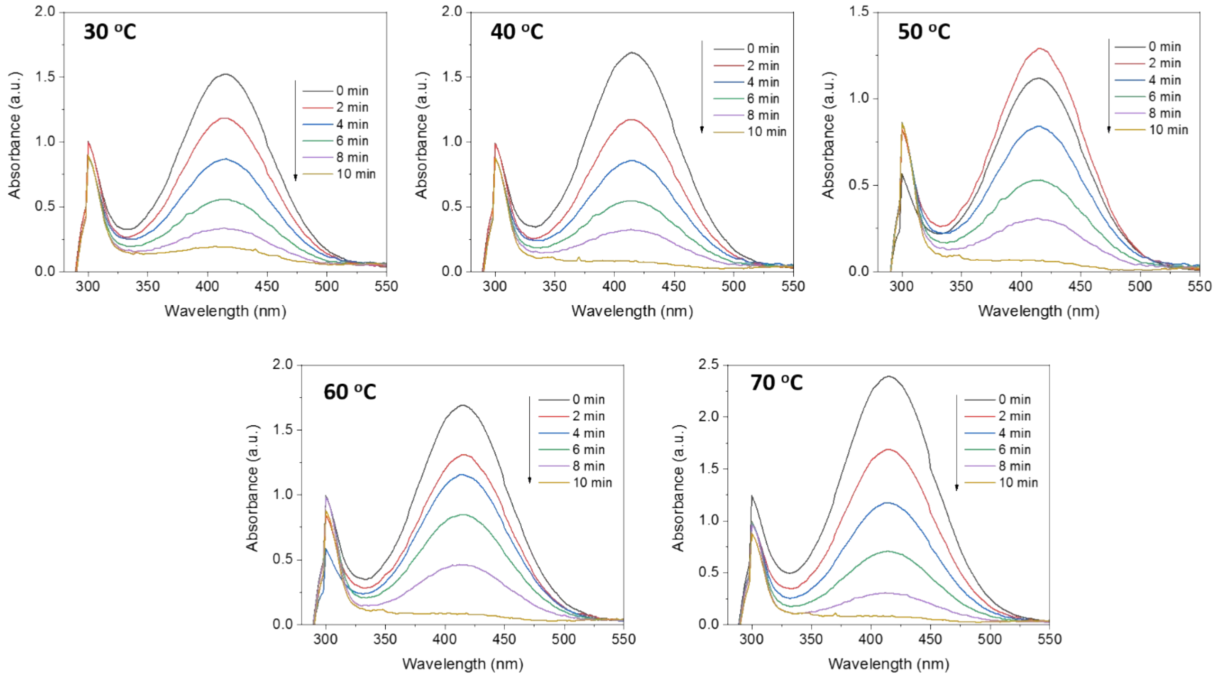

Fig. S1. UV-vis spectra for reduction of 2-NP at different temperatures

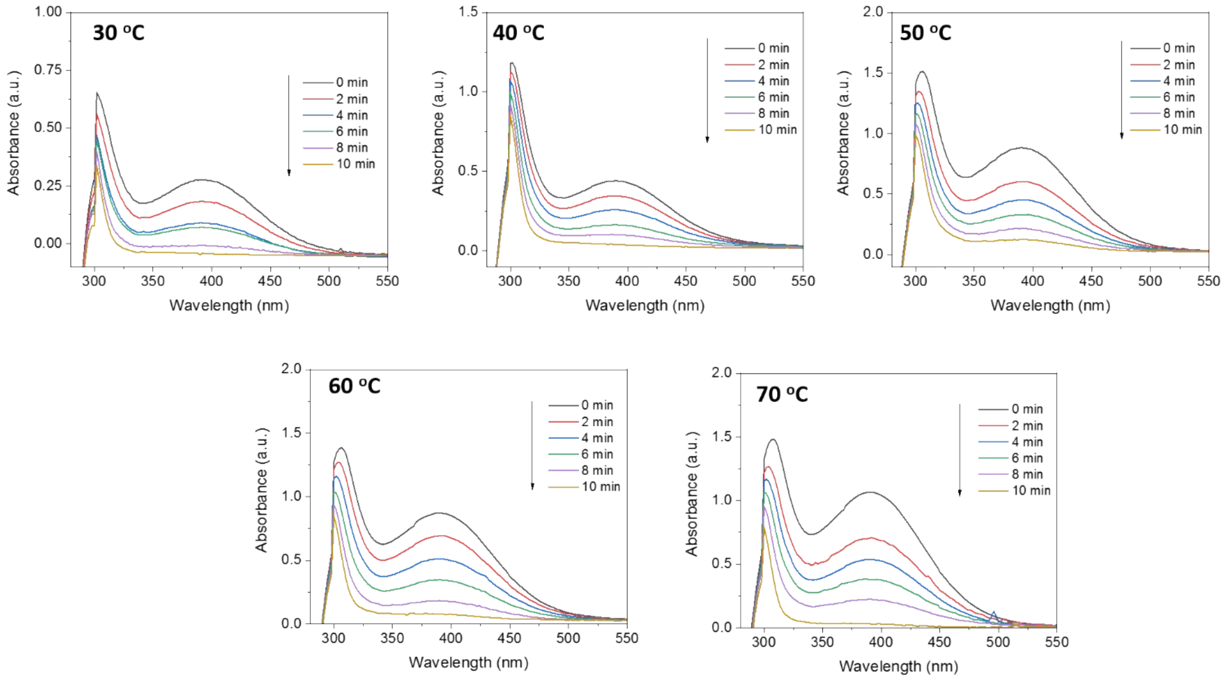

Fig. S2. UV-vis spectra for reduction of 3-NP at different temperatures

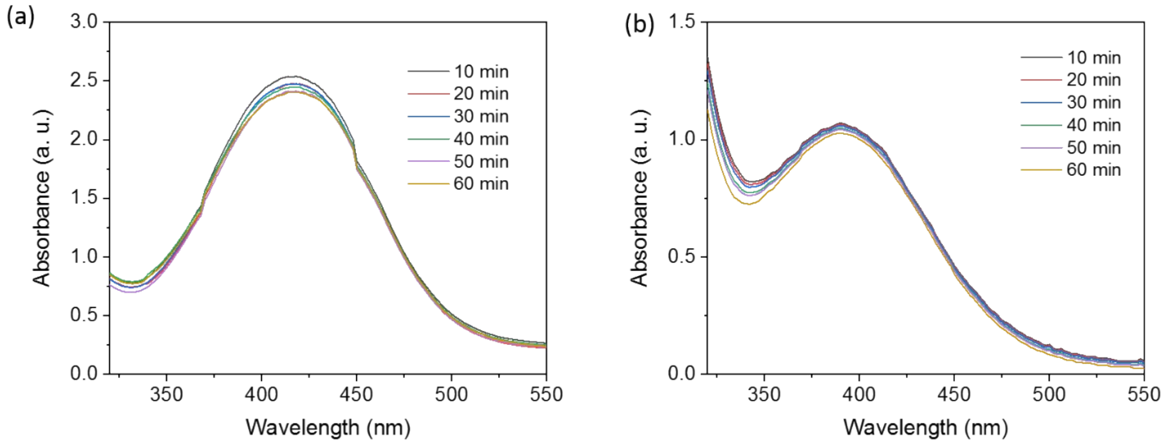

Fig. S3. UV-vis spectra of the $NaBH_4$ reduction of 2-NP (a) and 3-NP (b)

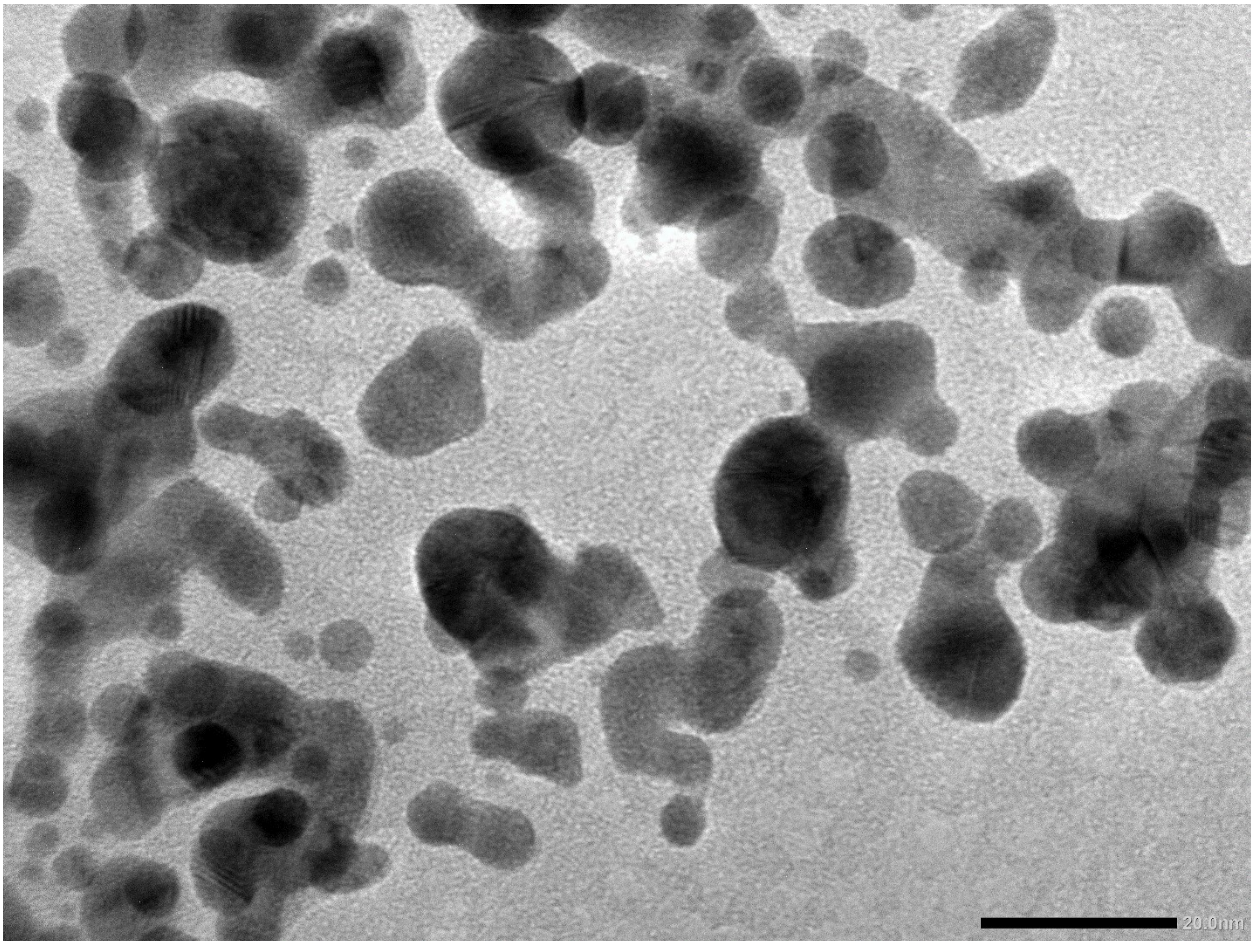

Fig. S4. TEM image of SG-AuNPs after five recycles for the catalytic degradation of 2-NP

Table S1. Comparison of the catalytic reduction of 2-NP and 3-NP by AuNPs/$NaBH_4$ system

**Catalyst**	**Average particle size (nn)**	**Pollutant**	**Rate constant (x 10^{-3} sec^{-1})**	**Ref.**
Seaweed *Lobophora variegate* - AuNPs	11.69	2-NP	1.2	1
		3-NP	4.5	
Corn-cob-AuNPs	35	2-NP	3	2
		3-NP	8	
Codonopsis pilosula root-AuNPs	20	2-NP	1.2	3
		3-NP	4.1	
Fungus *Trichoderma sp*. WL-Go-AuNPs	9.8	2-NP	7.4	4
		3-NP	10.3	
Magnusiomyces ingens LH-F1-AuNPs	28.3	2-NP	7.1	5
		3-NP	14.3	
SG-AuNPs	7.5	2-NP	3.0	This study
		3-NP	1.4	

References

1 P. Kaithavelikkakath Francis, S. Sivadasan, A. Avarachan and A. Gopinath, *Part. Sci. Technol.*, 2020, **38**, 365–370.

2 V.-D. Doan, V.-S. Luc, T. L.-H. Nguyen, T.-D. Nguyen and T.-D. Nguyen, *Environ. Sci. Pollut. Res.*, 2020, **27**, 6148–6162.

3 V.-D. Doan, B.-A. Huynh, T.-D. Nguyen, X.-T. Cao, V.-C. Nguyen, T. L.-H. Nguyen, H. T. Nguyen and V. T. Le, *J. Nanomater.*, 2020, **2020**, 1–18.

4 Y. Qu, X. Li, S. Lian, C. Dai, Z. Jv, B. Zhao and H. Zhou, *IET Nanobiotechnology*, 2019, **13**, 12–17.

5 Y. Qu, S. You, X. Zhang, X. Pei, W. Shen, Z. Li, S. Li and Z. Zhang, *Bioprocess Biosyst. Eng.*, 2018, **41**, 359–367.
